# Energy metabolism in glioblastoma stem cells: PPARα a metabolic adaptor to intratumoral microenvironment

**DOI:** 10.18632/oncotarget.19086

**Published:** 2017-07-07

**Authors:** Alessia Fidoamore, Loredana Cristiano, Chiara Laezza, Renato Galzio, Elisabetta Benedetti, Benedetta Cinque, Andrea Antonosante, Michele d’Angelo, Vanessa Castelli, Maria Grazia Cifone, Rodolfo Ippoliti, Antonio Giordano, Annamaria Cimini

**Affiliations:** ^1^ Department of Life, Health and Environmental Sciences, University of L’Aquila, L’Aquila, Italy; ^2^ Institute of Endocrinology and Experimental Oncology, IEOS, CNR, Naples, Italy; ^3^ Sbarro Institute for Cancer Research and Molecular Medicine, Department of Biology, Temple University, Philadelphia, Pennsylvania, USA; ^4^ National Institute for Nuclear Physics (INFN), Gran Sasso National Laboratory (LNGS), Assergi, Italy; ^5^ Department of Medicine, Surgery and Neuroscience, University of Siena, Siena, Italy

**Keywords:** tumor stem cells, metabolism, PPARs

## Abstract

Glioblastoma (GB), the most-common cancer in the adult brain, despite surgery and radio/ chemotherapy, is to date almost incurable. Many hypoxic tumors, including GB, show metabolic reprogramming to sustain uncontrolled proliferation, hypoxic conditions and angiogenesis. Peroxisome Proliferator-activated Receptors (PPAR), particularly the α isotype, have been involved in the control of energetic metabolism. Herein, we characterized patient-derived GB neurospheres focusing on their energetic metabolism and PPARα expression. Moreover, we used a specific PPARα antagonist and studied its effects on the energetic metabolism and cell proliferation/survival of GB stem cells. The results obtained demonstrate that tumor neurospheres are metabolically reprogrammed up-regulating glucose transporter, glucose uptake and glycogen and lipid storage, mainly under hypoxic culture conditions. Treatment with the PPARα antagonist GW6471 resulted in decreased cell proliferation and neurospheres formation. Therefore, PPARα antagonism arises as a potent new strategy as adjuvant to gold standard therapies for GB for counteracting recurrences and opening the way for pre-clinical trials for this class of compounds. When tumor neurospheres were grown in hypoxic conditions in the presence of different glucose concentrations, the most diluted one (0.25g/L) mimicking the real concentration present in the neurosphere core, PPARα increase/PPARγ decrease, increased proliferation and cholesterol content, decreased glycogen particles and LDs were observed. All these responses were reverted by the 72 h treatment with the PPARα antagonist.

## INTRODUCTION

Glioblastoma (GB) is the most common primary brain tumor with poor prognosis. Its invasive nature and its resistance to therapy result in a very high rates of recurrence [1]. GB stem cells (GSCs) have been indicated as responsible for drug resistance and relapse. GSCs reside in intratumoral perivascular and necrotic/hypoxic niches [2]. Hypoxia has recently emerged as a major factor influencing tumor proliferation and malignant progression. Although some of the effects of hypoxia negatively impact tumor cell growth, they may, antithetically, lead to hypoxia-driven responses that enhance malignant progression and aggressiveness, ultimately resulting in increased resistance to therapy and poor long-term prognosis. In GB, the malignant progression associated with tumor hypoxia appears to be mediated by several mechanisms, including changes in gene expression, inactivation of suppressor genes or activation of oncogenes, genomic instability, loss of apoptotic potential, induction of angiogenesis and invasive phenotypical changes [3-4]. Cellular responses to hypoxia are commonly regulated by the HIFs, a family of transcriptional factors upregulating the transcription of numerous hypoxia-inducible genes. HIF proteins function as master regulators of oxygen levels in cells through conformational changes in response to varying O_2_ concentrations, and, in gliomagenesis, as key regulatory elements in a variety of physiological and pathological responses to hypoxia, regulating genes involved in tumor progression, angiogenesis, drug resistance and GSC phenotype maintenance [5]. HIF-1α induces the expression of genes that trigger the tumor metabolic shift, promoting the increase of glucose uptake, the expression of glycolytic enzymes, the anaerobic glycolytic pathway of ATP generation,, and lactate production; it regulates pyruvate metabolism in both hypoxic and normoxic cells, increasing the production of precursors needed for cell growth [3, 6]. HIF-1α also controls fatty acid and glycogen synthesis, inducing the expression of the enzymes required to convert glucose to glycogen, including hexokinase (HK1 or HK2), phosphoglucomutase1 (PGM1), UDP-glucose pyrophosphorylase (UGP2), glycogen synthase (GYS1), glycogen branching enzyme (GBE1), as well as the gene encoding PPP1R3C, which activates GYS1 and inhibits liver-type glycogen phosphorylase (PYGL), that breaks down glycogen [7]

Although little is known about the bioenergetics resetting of CSCs, it would appear that a direct link exists between the occurrence of a metabolic switch from OXPHOS to aerobic glycolysis and the occurrence and maintenance of CSC cellular states [8]. A direct link between glucose metabolism and cancer stem/initiating cells has similarly been established in GB cancer tissues [9]. It has been suggested that certain bioenergetic features such as the Warburg effect can no longer be viewed as the single metabolic entity shared by all the cancer tissues, but rather as the archetypical aspect of the undifferentiated state, owned by CSCs [8]. Thus, the permanent shift toward a Warburg effect might be an essential contributory cause to cancer and might represent the central link between the genetic/epigenetic instability and the CSC theory, considering that a characteristic and essential feature of each neoplasm is the lack of differentiation [10].

Analyzing the general lipid composition of human gliomas, it was shown that total lipids accounted for 15-35% of the dry weight of the tissue with about 25% cholesterol not found in its free form while, in normal brain tissue, all cholesterol was in its free form [11]. The free fatty acid level, including polyunsaturated fatty acids, particularly linoleic acid, was higher in glioma, meningioma, neurinoma and carcinoma than normal brain tissue, suggesting that gliomas developed an altered metabolism for fatty acids. Another important biosynthetic process within lipid metabolism, up-regulated in GB, is the mevalonate pathway, which is known to facilitate the synthesis of cholesterol. Cholesterol esters, formed by the esterification of cholesterol with long-chain fatty acids, have been shown to be present only in high-grade gliomas [12-13]. However, why neoplastic tissues form and accumulate cholesterol esters, and how tumor cells utilize this class of lipids is unknown. Given that the levels of free cholesterol are strictly regulated by negative feedback mechanisms, formation of cholesterol esters could be the strategy that glioma cells use to store cholesterol. When cells require cholesterol, cholesterol esters could quickly release cholesterol for cell growth or survival. Therefore, considering that cholesterol esters are absent in healthy brain tissues, preventing their utilization may constitute a possible therapeutic strategy to inhibit malignant glioma growth [14]. Although it is generally accepted that tumor cells, particularly CSC, modify their glucose and lipid metabolism inside tumor microenvironment, the specific metabolic pathways and their regulation are still poorly understood. Owing to their crucial roles in energetic metabolism, many Authors have considered the role of the peroxisome proliferator-activated receptors (PPARs) in tumorigenesis, with some studies implicating these factors in the promotion and development of cancer, while others presenting evidence for an antitumorigenic role for these receptors [15]. In brain tumors PPARs have been demonstrated to play a role in controlling growth and malignancy [16-22] as well as growth and expansion of brain GSC [23]. PPARα activation increases proliferation in breast cancer cell lines and in renal cell carcinoma cell line [24-25] and causes liver cancer in rodents, while PPARα null-mice are shown to be resistant to hepatocarcinogenic effects of PPARα agonists [26]. Despite these findings, its function as tumor suppressor or inducer in cancers is still uncertain and may be related to cancer/cell type and/or specific microenvironment of the tumor.

On the basis of the reported findings, this work was focused on the possible correlation between the peculiar energetic metabolism of GSCs and PPARs. In particular, we evaluated the expression of PPARα and γ and the levels of proteins implicated in glucose and lipid metabolism in GSC, obtained from bioptic GB specimens and grown in normoxia and hypoxia, focusing the attention on some proteins crucial for glycolytic and cholesterol pathways and for glycogen and lipid storage.

Our results allow to positivly correlate PPARα to proliferation, survival and resistance of GSCs, through its regulation of glucose and lipid metabolism and demonstrate that a specific synthetic antagonist of PPARα, GW6471, inhibits these effects. The specific synthetic PPARα antagonist, GW6471 favours and reinforces the binding of co-repressors to the transcription factor [27], thus inhibiting its activity. The study of the effect of this antagonist on GSCs allows to elucidate the role of PPARα in their metabolism and to propose this molecule as potential drug specifically targeting these cells.

## RESULTS

### GSCs characterization

**Figure 1:**
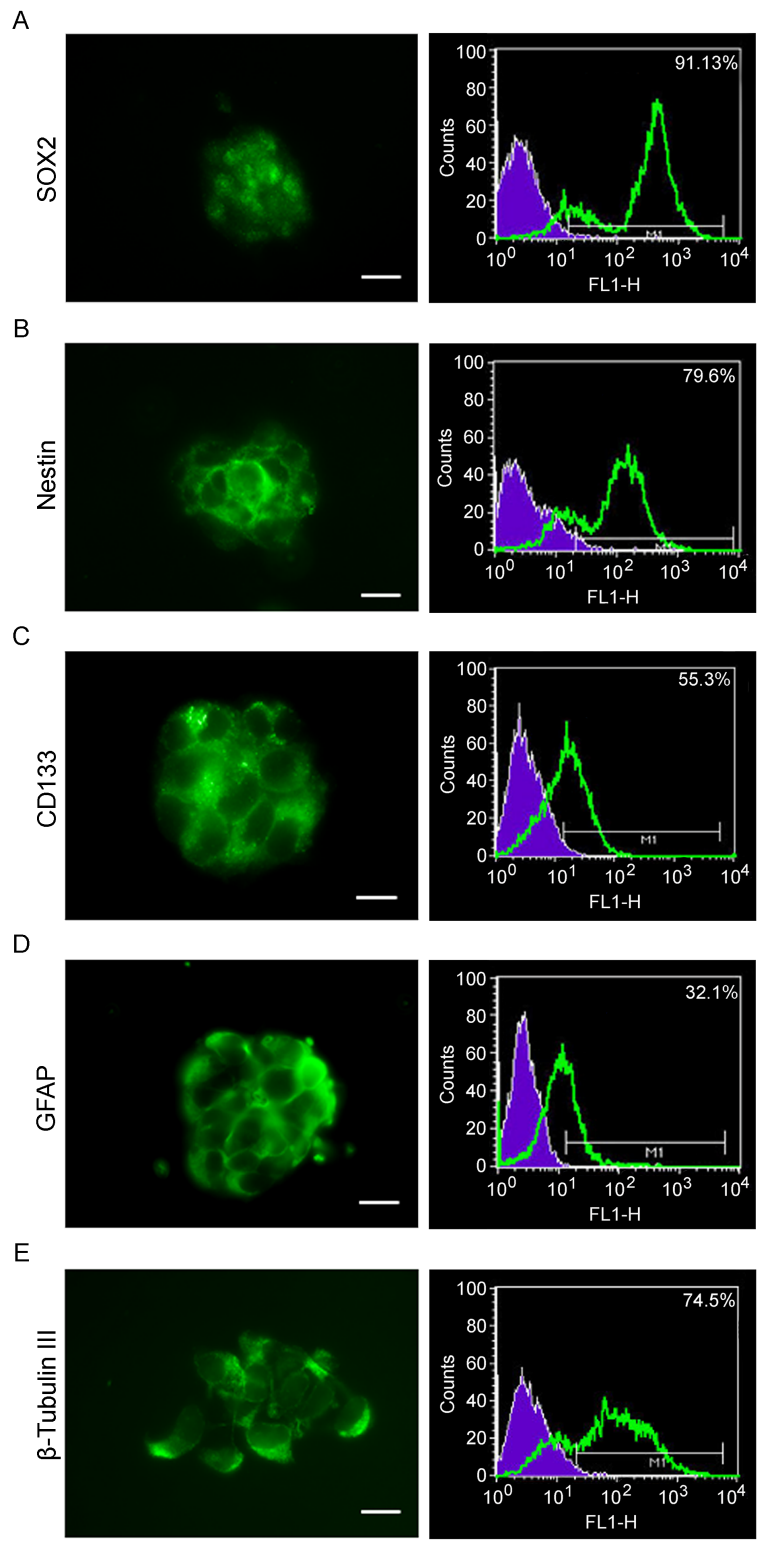
Stemness markers and multipotency of GSCs GSCs were characterized by cytofluorimetry and immunofluorescence. **A.**-**E.** Neurosphers characterization for stemness markers such as SOX2, Nestin, CD133, GFAP and β-tubulin III, show that GScs express all the stemness markers as well as the malignancy marker β-tubulin III. Bar = 25µm.

GSCs were characterized by the expression of stemness markers (Figure 1), such as SOX2 (A), nestin (B), CD133 (C), GFAP (D) by immunofluorescence and cytofluorimetric assay. Moreover, the malignancy marker β-tubulinIII (E) was also assessed. The results obtained, in agreement with the current literature, demonstrate that our cells were GSCs.

### Expression of PPARα and γ in GSCs

**Figure 2:**
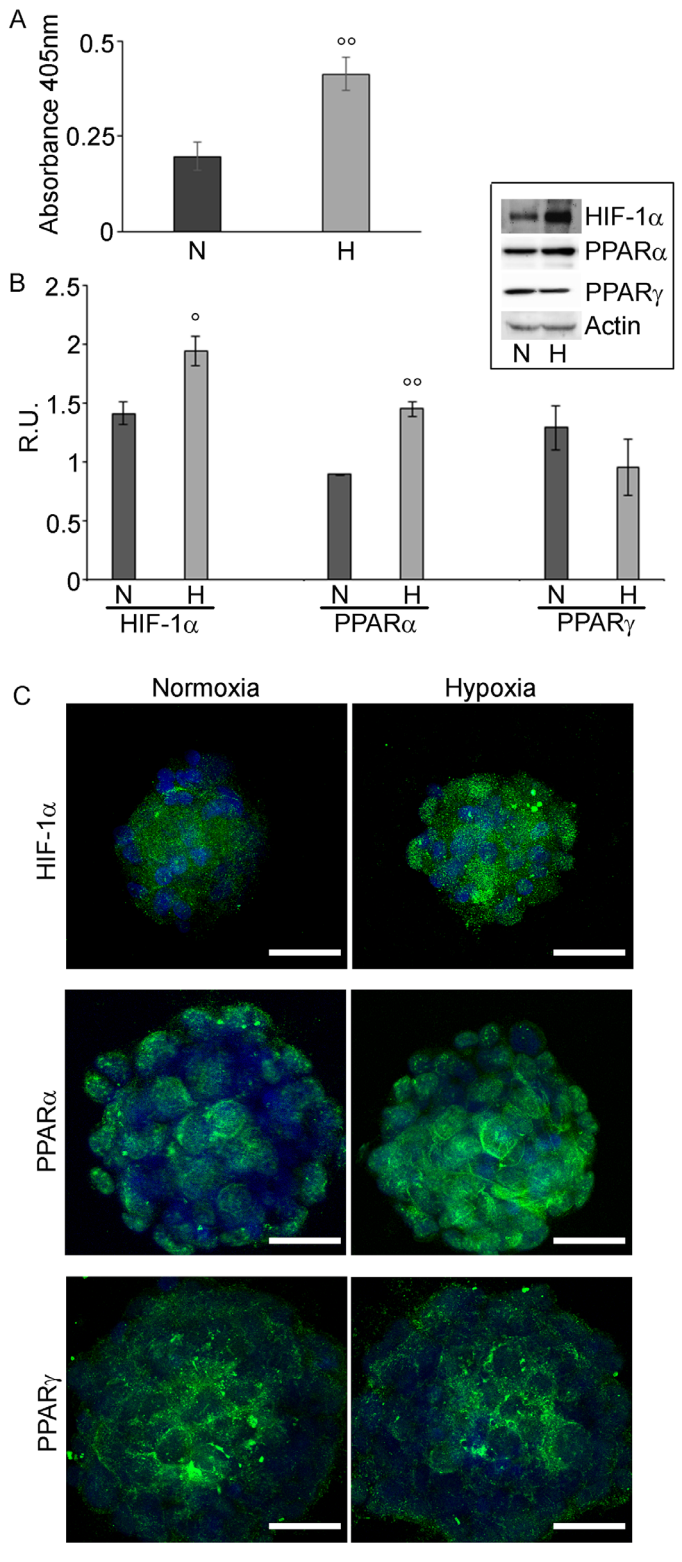
GSCs Proliferation assay and HIF-1α and PPARα and γ expression in GSCs **A.** BrdU assay in normoxia (N) and in hypoxia (H) of GSCs shows that hypoxic cells are more proliferating than normoxic ones.. Data are means ± SD of 3 different experiments. °°, *p* < 0.001, hypoxia *vs* normoxia. **B.** Western blotting analysis for HIF-1α, PPARα and γ in normoxic and hypoxic GSCs. In hypoxic conditions, a higher expression of HIF-1α and PPARα is observed, while PPARγ appears not significantly different. Data are means ± SD of 3 different experiments. °, *p* < 0.01, °°, *p* < 0.001, hypoxia *vs* normoxia. **C.** Immunofluorescence for HIF-1α, PPARα and γ in normoxic and hypoxic GSCs. In agreement with the western blotting results, HIF-1α and PPARα appeared highly expressed, mainly at nuclear level. Nuclei are stained with Dapi. Bar = 25µm.

The cell proliferation assay (Figure 2A) showed that hypoxia significantly enhances GSCs proliferation. Moreover, the hypoxic cells exhibited higher levels of HIF-1α protein (Figure 2B), most probably active, being mainly localized to the nucleus (Figure 2C). PPARα and γ expression was evaluated by western blotting analysis and immunofluorescence (Figures 2B and 2C). PPARα, showed a cytoplasmic and nuclear localization and was significantly upregulated in hypoxic cells, while PPARγ was localized mainly in the cytoplasm and in hypoxia it appears at lower levels.

### Hypoxia promotes accumulation of glycogen and LDs

**Figure 3:**
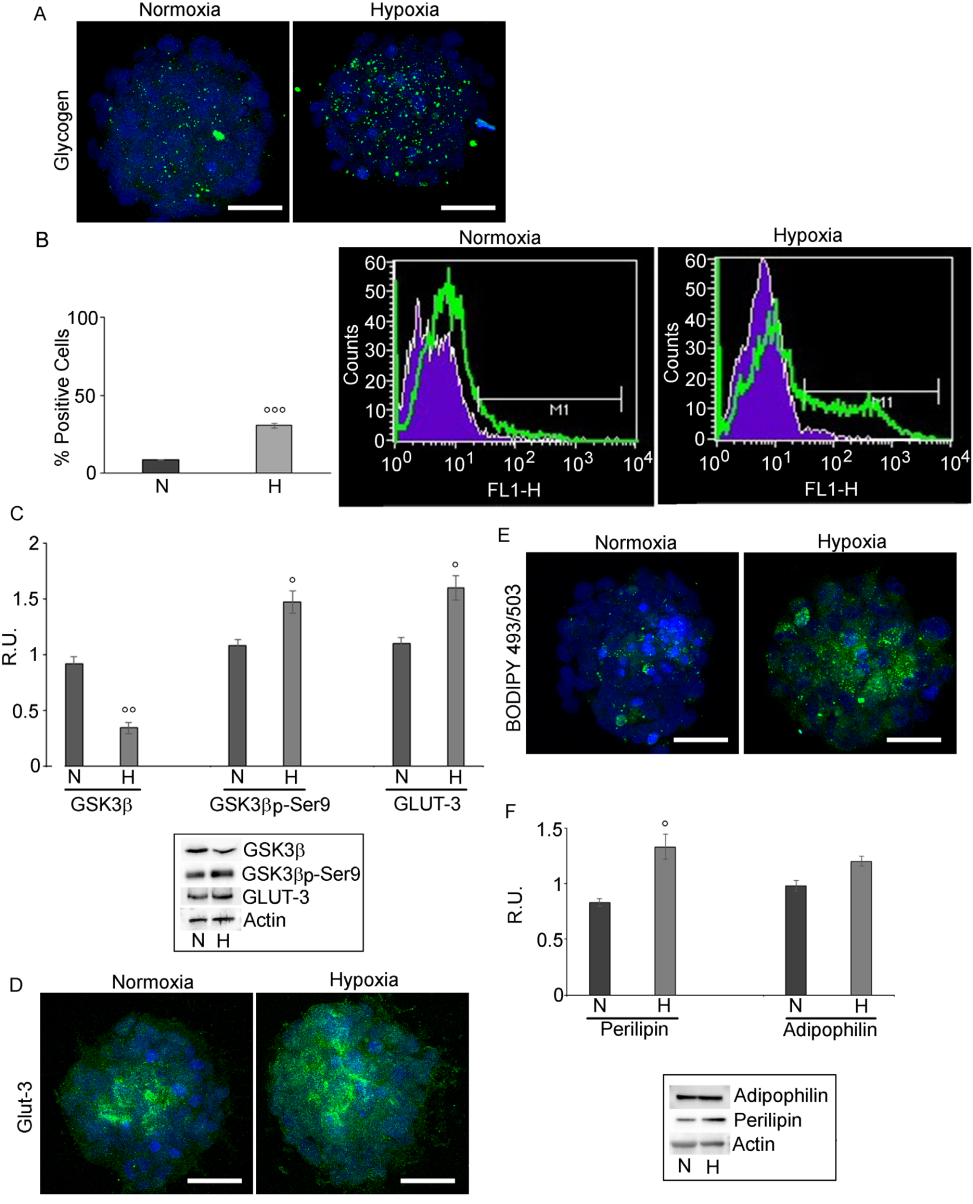
Glycogen and LD in GSCs evaluated by confocal microscopy and cytofluorimetry **A.** glycogen localization in normoxic and hypoxic GSCs shows that glycogen is highly present in hypoxic conditions . Nuclei are stained with Dapi. Bar = 25µm. **B.** Glycogen evaluation by FACS analysis confirmed the higher presence of glycogen in hypoxic neurospheres. Data are means ± SD of 3 different experiments. °°°, *p* < 0.0001 hypoxia *vs* normoxia. **C.** Western blotting analysis for GSK3β, p-GSK3β-ser9 and GLUT-3 shows a decrease of the active GSK3β paralleled by an increase of the inactive form p-GSK3β-ser9. GLUT-3 increases in hypoxia. Data are means ± SD of 3 different experiments. °, *p* < 0.01, °°, *p* < 0.001 hypoxia *vs* normoxia **D.** GLUT-3 immunofluorescence in normoxic and hypoxic GSCs confirmed the higher presence of GLUT-3 in hypoxic GSCs. Nuclei are stained with Dapi. Bar = 25µm. **E.** BODIPY 493/503 staining of LD in N and H conditions show the higher LD content in hypoxic neurospheres. Nuclei are stained with Dapi. Bar = 25µm. **F.** Western blotting analysis for perilipin and adipophilin in normoxic and hypoxic GSCs shows that perilipin is significantly increased in hypoxia, while adipophilin is not significantly modulated. Data are means ± SD of 3 different experiments. °, *p* < 0.01 hypoxia *vs* normoxia.

The hypoxic challenge increased glycogen as shown by specific immunofluorescence (Figure 3A). FACS analysis (Figure 3B) showed that glycogen-positive cells were more numerous in hypoxia than in normoxia. In parallel, a decrease, at protein level, of the active form of GSK3β, responsible for the repression of the glycogen synthase and the increase of its inactive phosphorylated form (Figure 3C) was observed, thus indicating increase of glycogen synthesis in hypoxia. In addition, the expression of the glucose transporter, GLUT-3, specifically present in brain tumor initiating cells, [9] was examined. The western blotting and immunofluorescence results (Figures 3C and 3D) showed that GLUT-3, mainly localized in the core of the normoxic neurospheres, appeared upregulated and ubiquitously distributed in hypoxic spheres.

LD accumulation was observed in adult neural stem cells [28], in colorectal cancer stem cells [29], in glioma cells, where they have been correlated to the malignancy grade [30, 31]. LDs, detected by BODIPY 493/503 (Figure 3E), were mainly localized in the core of both normoxic and hypoxic neurospheres and strongly increased in hypoxic condition. In parallel, perilipin, a LD membrane protein, crucial for the regulation of lipids storage/mobilization [32] increased significantly in hypoxia, while adipophilin, other component of LD membrane, was not significantly affected (Figure 3F).

### Effect of PPARα antagonist GW6471 on normoxic and hypoxic GSCs

**Figure 4:**
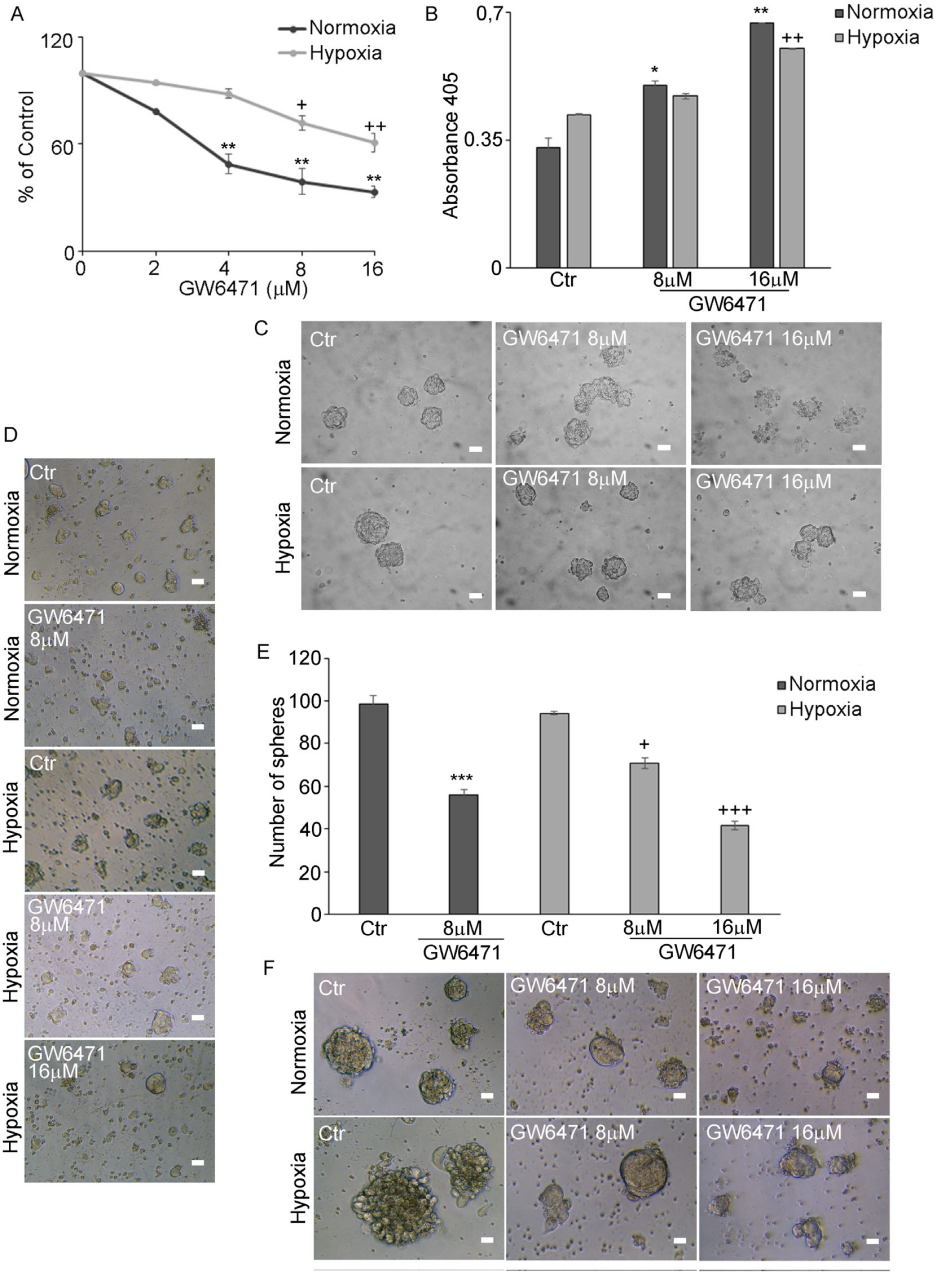
GW6471 effect on normoxic and hypoxic neurospheres **A.** BrdU assay in N and H neurospheres treated with 2-16µM GW6471 shows that normoxic cells are significantly affected in the range 4-16 µM, while the hypoxic cells appeared more resistant being decreased in the range 8-16 µM. Data are means ± SD of 3 different experiments. +, *p* < 0.01; ++, *p* < 0.001, hypoxia *versus* respective control; **, *p* < 0.001, normoxia *versus* respective control. **B.** Apoptosis assay shows that in normoxic cells apoptosis increased at 8-16 µM GW, while in hypoxia only 16 µM was effective in inducing cell death.. Data are means ± SD of 3 different experiments. ++, *p* < 0.001, hypoxia *versus* respective control; *, *p* < 0.01; **, *p* < 0.001, normoxia *vs* the respective control. **C.** Phase contrast microscopy of control and treated neurospheres in hypoxic and normoxic conditions. Bar = 70μm. **D.** Phase contrast microscopy of dissociated single cells from neurospheres after the treatment in hypoxia and normoxia. Bar = 70μm. **E.** Number of spheres formed after the treatment of dissociated single cells. Data are means ± SD of 3 different experiments. ***, *p* < 0.001 normoxia with respect to the respective control condition; +, *p* < 0.01; +++, *p* < 0.0001, hypoxia *vs* respective control condition. **F.** Phase contrast microscopy of neurospheres formed after antagonist treatment and monitored for further 7 days *in vitro*. Bar = 70μm.

A specific synthetic PPARα antagonist, GW6471, that favours and reinforces the binding of co-repressors to the transcription factor [27], inhibiting its activity, highly specific by the binding the peroxisome proliferator-activated receptor-α ligand-binding domain bound, was then used. The dose-effect curve (2-16µM), (Figure 4A), demonstrated that the antagonist determined, after 72hr, a progressive dose-dependent reduction of cell proliferation both in normoxic and hypoxic conditions. In normoxia the effect was significant already at 4μM, in hypoxia the drug affected cell proliferation, although to a lesser extent, starting from 8μM dose and significantly at 16μM. It is worth noting that this compound has been already tested on human normal renal epithelial cells at 25 µM, where it did not cause any toxicity. [25].

In addition to its cytostatic effect, GW6471 promoted apoptosis (Figure 4B). The increase of cell death was dose-dependent in both conditions, although hypoxic cells were more resistant to the treatment. The analysis of cell morphology, by phase contrast microscopy (Figure 4C) showed that GW6471 induced a disaggregation of normoxic spheres, while the hypoxic ones, although reduced in size, resulted still compact also at higher doses,. The effect of the antagonist was evaluated also on the formation of neurospheres, performing the 72hr treatment on single dissociated stem cells. The phase contrast images of neurospheres (Figure 4D) and the analysis of the relative counts (Figure 4E) revealed that GW6471 inhibited the formation of the spheres at the different doses both in normoxia and hypoxia. In fact, 8µM in normoxia decreased the number of spheres to about 50%, while in hypoxia the same effect was observed only at 16µM, in agreement with the BrdU results. After 72hr of treatment, normoxic and hypoxic spheres were monitored for further 7 days *in vitro* and analysed by phase contrast microscopy (Figure 4F). In both conditions, neurospheres derived from the residual and resistant cells to the treatment, were smaller in size than their controls, suggesting that the cells that did not undergo apoptosis during the treatment, were however subjected to an arrest/reduction of cell proliferation.

### GW6471 treatment on glycogen and lipid content

**Figure 5:**
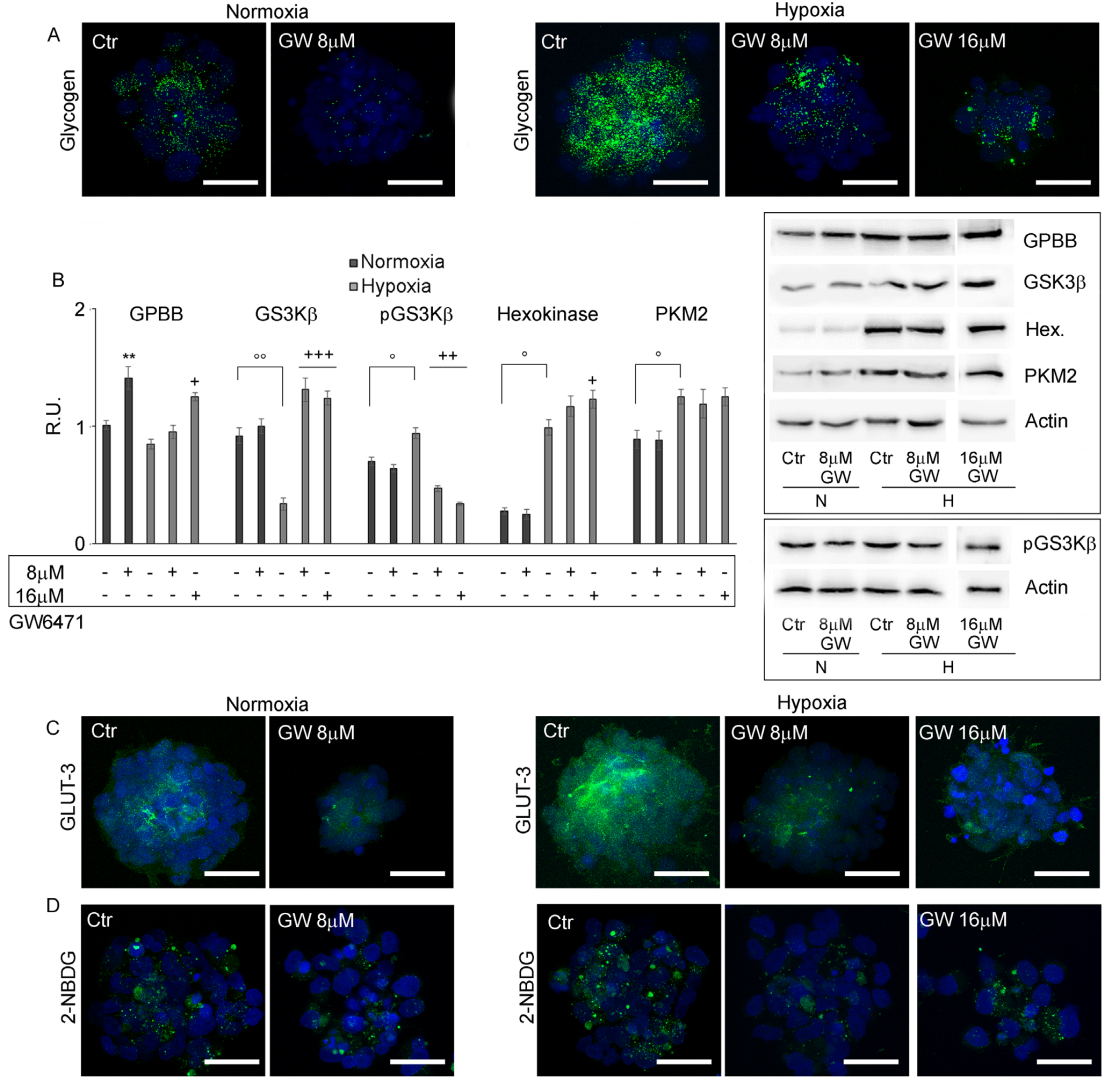
GW6471 effects on glycogen storage and metabolism of GSCs **A.** Glycogen levels in treated GSCs by confocal microscopy shows that glycogen is highly present in hypoxic neurospheres and that GW6471 is able to decrease glycogen both in normoxic and hypoxic neurospheres. Nuclei are stained with Dapi. Bar = 25µm. **B.** Western blotting analysis for GPBB, GSK3β, p-GS3Kβ-ser9, Hexokinase and PKM2. Data are means ± SD of 3 different experiments. +, *p* < 0.01, ++, *p* < 0.001, +++, *p* < 0.0001, hypoxia with respect to the respective control; °, *p* < 0.01, °°, *p* < 0.001, hypoxia *vs* normoxia. **, *p* < 0.001, normoxia *vs* the respective control. **C.** GLUT-3 immunofluorescence in treated GSCs in normoxic and hypoxic conditions shows that GLUT-3 is increased in hypoxia and that the treatment decreases the transporter both in normoxia and hypoxia. Nuclei are stained with Dapi. Bar = 25µm. **D.** Glucose uptake in treated GSCs by deoxy-glucose analog shows that the treatment was able to decrease glucose uptake. Nuclei are stained with Dapi Bar = 25µm.

**Figure 6:**
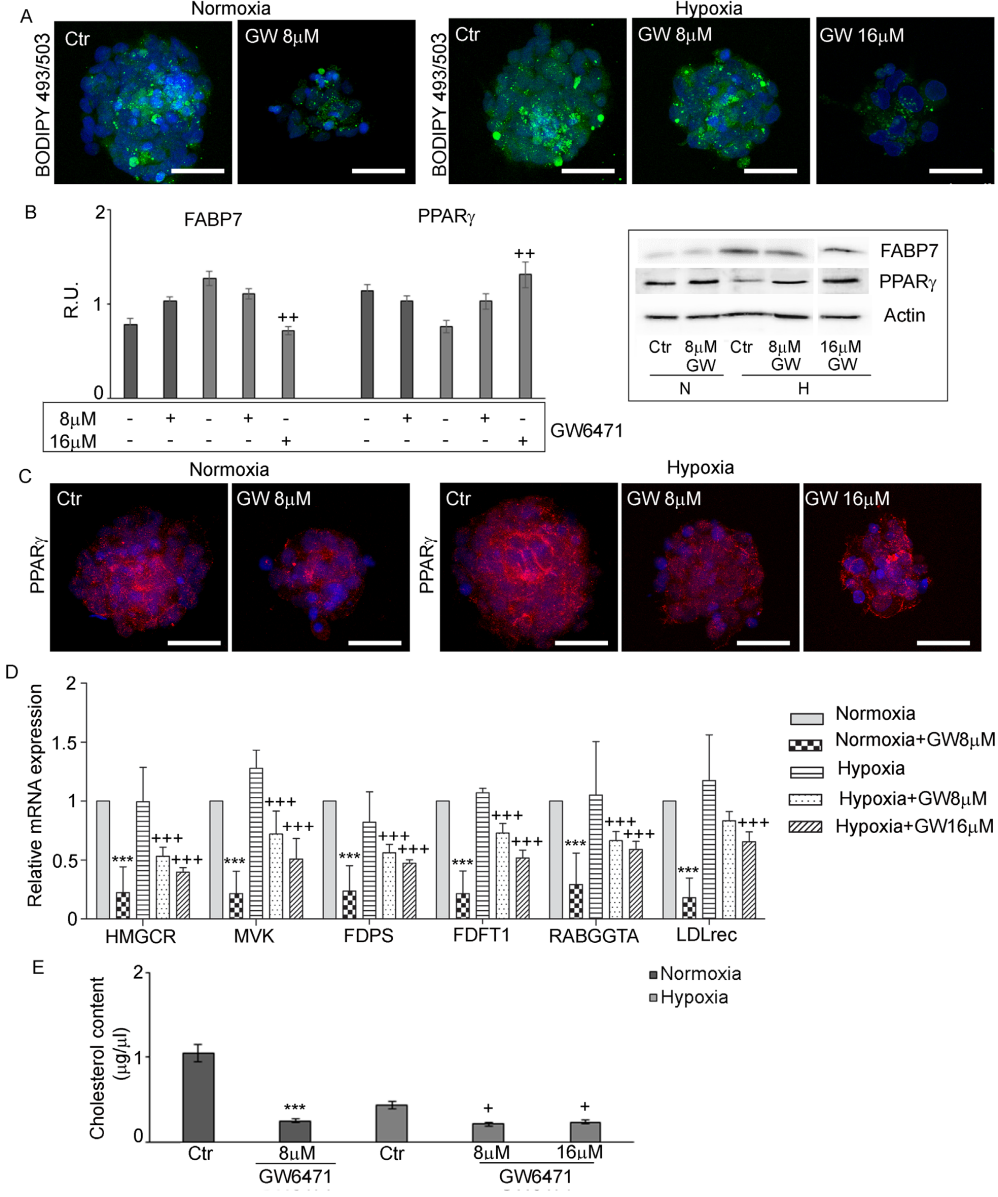
GW6471 effects on lipid droplets and cholesterol content in normoxic and hypoxic GSCs **A.** LD stained with BODIPY 493/503 in treated GSCs shows a significant decrease of LDs in both conditions. Nuclei are stained with Dapi. Bar = 25µm. **B.** Western blotting analysis for FABP7 and PPARγ shows that only in hypoxic condition at 16 µM, the treatment was able to decrease FABP7 and to increase PPARγ. Data are means ± SD of 3 different experiments. ++, *p* < 0.001 hypoxia *vs* the respective control **C.** PPARγ immunofluorescence in treated GSCs supports the western blotting results. Nuclei are stained with Dapi. Bar = 25µm. **D.** qRT-PCR analysis of the enzymes of the mevalonate pathway in treated GSCs. Data are means ± SD of 3 different experiments. +++, *p* < 0.0001 hypoxia *vs* the respective control,***,*p* < 0.0001, normoxia with respect to the respective control. **E.** Cholesterol content in treated GSCs shows a significant decrease of cholesterol content upon treatment in both conditions. Data are means ± SD of 3 different experiments. +, *p* < 0.01, hypoxia *vs* the respective control; ***, *p* < 0.0001, normoxia with respect to the respective control.

The levels of glycogen and lipid storage were investigated after antagonist treatment. Figure 5A shows that the inhibition of PPARα in normoxia with 8µM GW6471 determined a strong reduction of glycogen content in all cells of the spheres, while in hypoxia it induced a slight decrease of glycogen granules limited to the external layers of the neurospheres, while the cells in the core preserved their storage. The 16µM dosage in hypoxia resulted more effective in reducing the glycogen content also in the hypoxic core of the spheres. The analysis, by western blotting, of key enzymes involved in glycogenolysis and in glycolytic pathways (Figure 5B) demonstrated that glycogen phosphorylase (GPBB), a brain specific isoenzyme of the first rate-limiting step of glycogenolysis, responsible for the conversion of glycogen into glucose 1-phosphate, was significantly increased in the normoxic cells, after treatment, in agreement with the decrease of glycogen granules observed by immunofluorescence. In hypoxia, no variation of the protein at 8µM antagonist was observed, while it significantly increased at 16µM. It is worth noting, only in hypoxia, that the treatment with GW6471 both at 8 and 16µM promoted an increase of the expression of the active form of GSK3β as well as a reduction of its inactive phosphorylated form. It appears that, while in normoxia the reduction of glycogen content may be due mainly to an increase of glycogenolysis, in hypoxia it may be dependent on both the blockade of glycogen synthesis and on the increase of glycogen demolition. As regard glycolytic enzymes, hexokinase II and pyruvate kinase M2 (PKM2), highly present in proliferating tumor cells, they were significantly upregulated in hypoxia compared to normoxia, since they are HIF-1α target genes [7]; the treatment with GW6471 did not affect their protein levels in both conditions. In order to evaluate if the decrease of glycogen in the presence of the antagonist might be due to a reduction of glucose uptake, the expression of GLUT-3 (Figure 5C) and the ability of GSCs to incorporate a D-glucose analog (2-NBDG) (Figure 5D) were investigated. GLUT-3 was overexpressed in hypoxia and GW6471 decreased the fluorescence intensity for the transporter paralleled by a decrease of glucose intake in both normoxic and hypoxic conditions. As regard lipid metabolism, the amount of LD (Figure 6A), the expression of Fatty Acid Binding Protein 7 (FABP7) and PPARγ (Figure 6B) and its localization (Figure 6C) and the enzymes of cholesterol synthesis by qReal Time PCR (Figure 6D) were investigated under GW6471 treatment. We observed a strong reduction of LDs in treated cells both in normoxia and in hypoxia, at 8µM and 16µM, respectively. Moreover, FABP7, responsible for the uptake and transport of fatty acids, marker of GSCs [33] and known to be regulated by PPARα [34], was upregulated in hypoxia and significantly downregulated in this condition upon the antagonist treatment. The higher levels of FABP7 in hypoxia were paralleled by the accumulation of LDs in hypoxic cells (Figures 3E and 3F). Finally, the analysis by qRT-PCR of the enzymes of the mevalonate pathway (Figure 6D) showed that the mRNA levels for HMG-CoA reductase (HMGCR), Farnesyl diphosphate synthase (FDPS), Farnesyl diphosphate farnesyltransferase 1 (FDFT1) or squalene synthase and geranylgeranyltransferase1 (RABGGTa) did not show significant differences between normoxia and hypoxia, except for mevalonate kinase (MVK) that was upregulated in hypoxic condition. GW6471 remarkably reduced all mRNAs analyzed, both in normoxia and in hypoxia, irrespective of the dose. As regard the mRNA for low-density lipoprotein receptor (LDLR), the GW6471treatment induced a dramatic reduction in normoxia and a dose-dependent decrease in hypoxia. These results suggest that the treatment with the PPARα antagonist, by inhibiting the MVA pathway, may impair the prenylation of proteins involved in migration and invasion of tumor cells. In addition, it is worth noting that the downregulation of LDLR, upon treatment, may compromise cholesterol influx, important for membrane turnover and for composition of LDs. The analysis of cholesterol content (both free cholesterol and cholesteryl esters) (Figure 6E), showed that normoxic GSCs possess more cholesterol than hypoxic cells and, upon treatment, strong and significant reduction of its level was observed. In hypoxia, the inhibition of PPARα exerted similar effects with both 8 and 16 µM, reducing the cholesterol content to about 50% of its control.

### Modulation of PPARs upon GW6471

**Figure 7:**
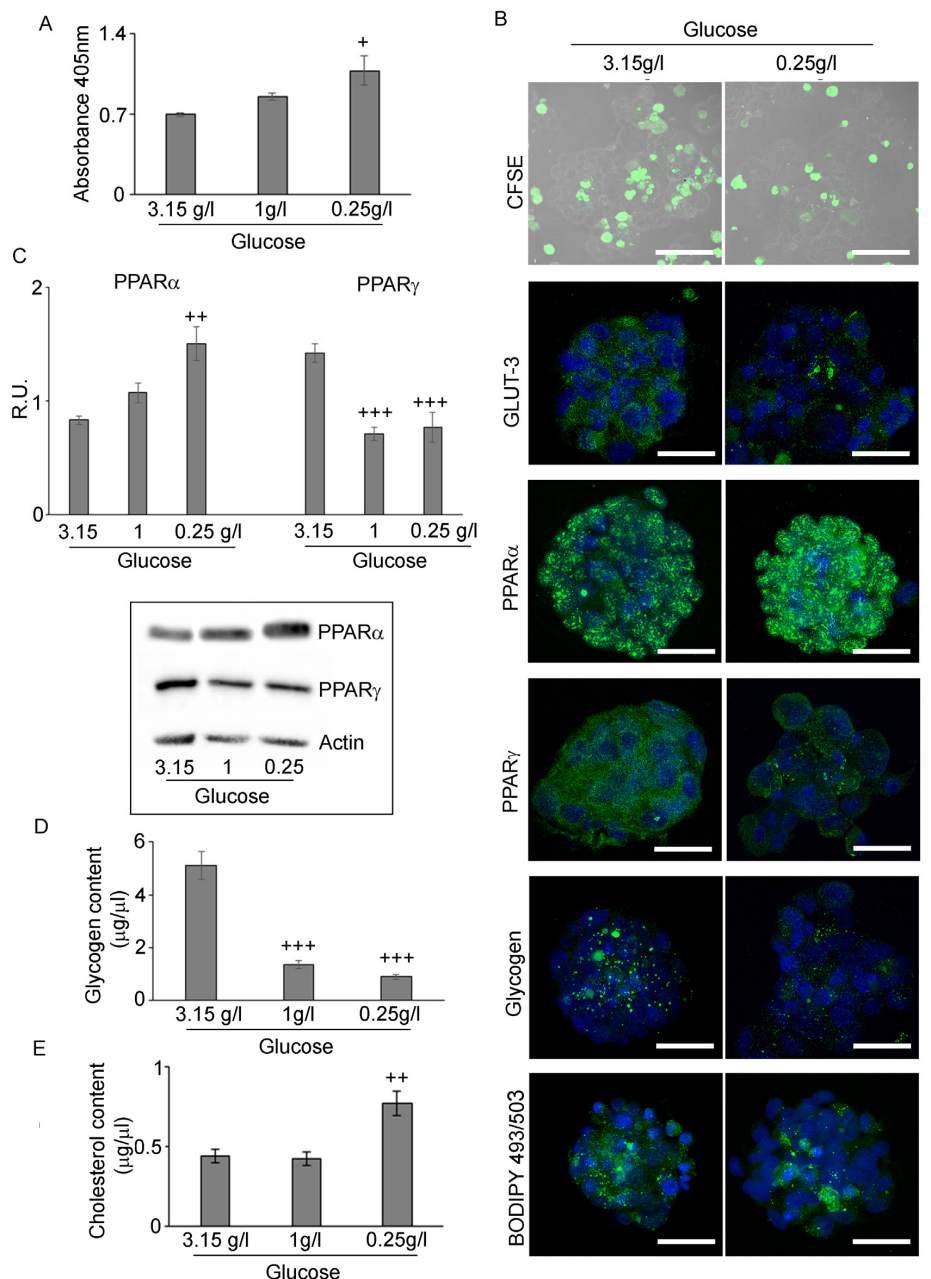
Hypoxic neurospheres grown in restricted glucose conditions **A.** BrdU assay of H neurospheres in medium containing 3,15g/L, 1g/L and 0,25g/L glucose shows that proliferation increase with glucose deprivation. Data are means ± SD of 3 different experiments. +, *p* < 0.01 *vs* high glucose **B.** CFSE assay (Bar = 50µm), GLUT3, PPARα and γ, glycogen and LD staining in the different glucose conditions shows that the increase of proliferation, PPARα and a decrease of PPARγ, glycogen and GLUT-3 (Bar = 25µm). **C.** Western blotting analysis of PPAR and γ confirmed the immunofluorescence data. Data are means ± SD of 3 different experiments. ++, *p* < 0.001; +++, *p* < 0.0001 with respect to high glucose condition. **D.** Glycogen assay in the different glucose conditions shows a reduction of glycogen content in glucose deprivation. Data are means ± SD of 3 different experiments. +++, *p* < 0.0001 with respect to high glucose condition. **E.** Cholesterol content in the different glucose conditions shows a significant increase of cholesterol content in glucose deprivation. Data are means ± SD of 3 different experiments. ++, *p* < 0.001 with respect to high glucose condition.

The evaluation of the expression pattern of PPARα and γ by western blotting and immunofluorescence, upon GW6471 treatment (Figures 7B and Supplementary Figure 1) showed that PPARα did not change its localization nor its protein levels in both conditions (Supplementary Figure 1), while PPARγ, mainly cytoplasmic in normoxic and hypoxic conditions, was predominantly nuclear after the treatment (Figure 6C). Moreover, at protein level, PPARγ, appeared upregulated in dose-dependent manner only in treated hypoxic neurospheres (Figure 6B).

### Effect of glucose concentrations on hypoxic GSCs

All the referred experiments were performed in the standard culture medium, containing 3.15g/L glucose. Since this concentration is higher than that existing inside the tumor, we checked the growth and the metabolic pattern of spheres at at 0.25g/L glucose, which is the concentration of the intratumoral hypoxic microenvironment [35]. In this condition, the effects of 16µM GW6471 treatment were re-evaluated. To this purpose, spheres were grown in a medium containing 3.15g/L (standard medium), 1g/L (physiologic blood levels) and 0.25g/L (interstitial fluid from solid tumor) glucose [9] in hypoxic conditions for 72hr. The BrdU assay showed that GSCs, in the presence of low glucose, were more proliferating than those grown in high glucose (Figure 7A), thus confirming that proliferation is inversely correlated to glucose concentration in the medium [35]. The division frequency of neurospheres, evaluated with the fluorescent dye carboxyfluorescein succimidyl ester (CFSE) and analyzed by confocal microscopy (Figure 7B), showed that, the number of cells retaining the dye was higher in high glucose medium, than those grown in low glucose, demonstrating higher proliferation rate in the latter condition. Moreover, it is worth noting that, in high glucose condition, CFSE-positive cells, resided mainly in the hypoxic core of spheres, indicating that the core is slowly-dividing with respect to the remaining cells of the spheres. In 0.25g/L glucose condition, the cells in the core were more proliferating, as demonstrated by their lower fluorescence intensity. The immunofluorescence and western blotting analysis for PPARα and γ showed that, while PPARα was significantly upregulated at 0.25g/L glucose, the γ isotype was strongly downregulated in both 1g/L and 0.25g/L glucose conditions (Figures 7B and 7C). These data suggest that the expression of these transcription factors is strictly related to the glucose concentration in the *medium* and that the high proliferation rate of GSCs grown in low glucose may be due to the upregulation of the α isotype. Interestingly, both glycogen and lipid storages were influenced by the glucose concentration, their levels being modulated at low glucose concentration, as demonstrated by immunofluorescence (Figure 7B) and by glycogen and cholesterol assays (Figures 7D and 7E). As regard glycogen, a strong decrease of the number of granules at both 1g/L and 0.25g/L glucose, mainly affecting the external cellular layers of neurospheres, was observed (Figure 7B). This result is consistent with the analysis of glycogen content (Figure 7D) that confirmed a significant decrease of glycogen at 1g/L and at 0.25g/L. Similarly to glycogen, LD content was influenced by the concentration of glucose in the medium (Figure 7B), decreasing at 0.25g/L glucose and remaining mainly accumulated in the core of the neurospheres. Interestingly, the cholesterol assay (Figure 7E) showed that GSCs grown in low glucose have higher levels of cholesterol than those cultured in high glucose. Since the protein levels of FABP7 did not change in these conditions (data not shown) while PPARα significantly increased, and PPARγ even decreased, we hypothesize that the reduction of LDs at 0.25g/L may be due to increased acyl-CoA-β-oxidation with production of acetyl-CoA to be utilized for the synthesis of cholesterol. Moreover, in this context, it is worth-recalling that, besides acyl-CoAβ oxidase, also HMG-CoA synthase, leading to increased HMG-CoA synthesis, is a PPARα target gene, [36].

### Effect of GW6471 in low glucose conditions

**Figure 8:**
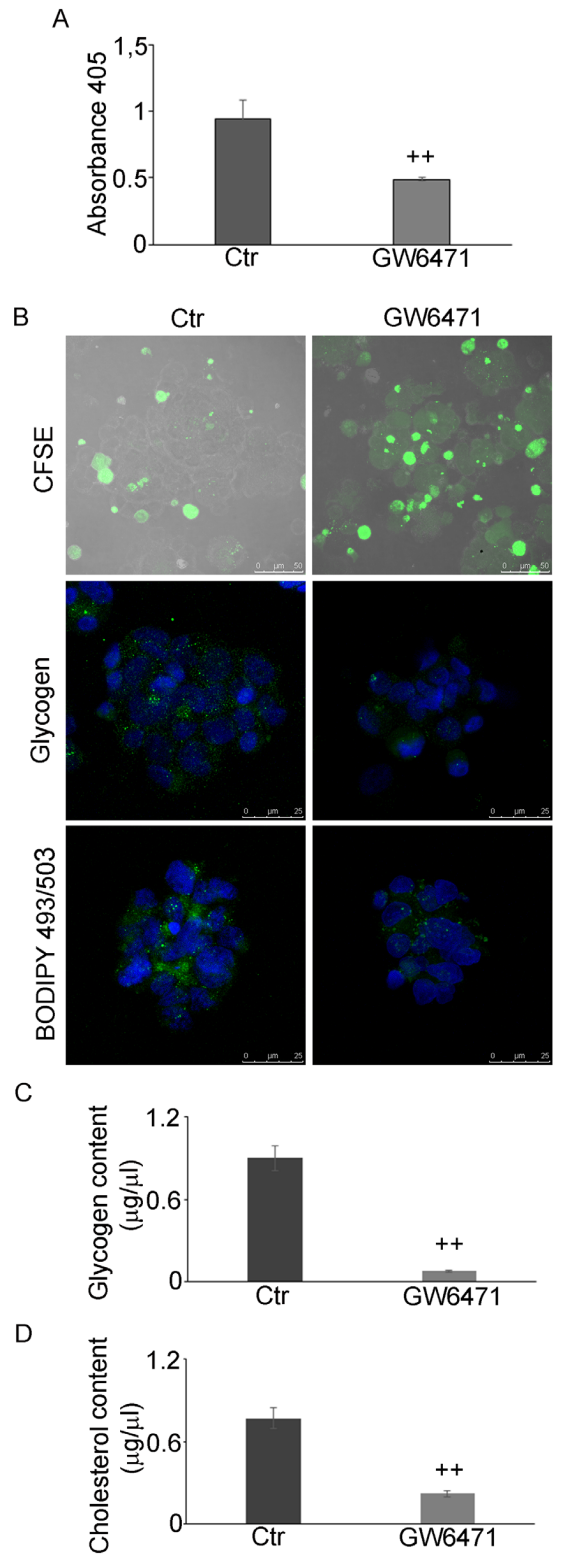
GW6471 effects on proliferation, glycogen and LD storage under glucose shortage **A.** BrdU assay of hypoxic neurospheres grown in 0.25g/L glucose and treated with 16µM antagonist shows a significant decrease of proliferation upon treatment. Data are means ± SD of 3 different experiments. ++, *p* < 0.001 *vs* control condition (not treated). **B.** CFSE assay (Bar = 50µm) and glycogen and LD staining shows a decrease of proliferation, glycogen and LD upon treatment (Bar = 25µm). **C.** Glycogen content analysis shows a significant decrease of glycogen upon treatment. Data are means ± SD of 3 different experiments. ++, *p* < 0.001 *vs* control condition (not treated). **D.** Cholesterol content decreases upon treatment. Data are means ± SD of 3 different experiments. ++, *p* < 0.001 *vs* control condition (not treated).

Finally, we studied the effect of 16µM GW6471 in low glucose conditions (0.25 g/L). The BrdU results (Figure 8A) showed that 16µM GW6471 induced an arrest of cell proliferation to about 50% of control, demonstrating that the drug is effective, as in high glucose conditions. The CFSE assay (Figure 8B), showing that treated cells retained more dye than controls, confirms that they were delayed and/or arrested in their proliferation. Particularly, we observed a gradient of staining intensity, ranging from cells with a more diluted staining, (low rate of proliferation) to those displaying the highest fluorescence intensity, corresponding to cell cycle-arrested or apoptotic cells.. Finally, in addition to its effect on cell proliferation rate, PPARα antagonist induced a further decrease of glycogen, LDs (Figure 8B) and glycogen and cholesterol content (Figures 8C and 8D), thus indicating that GSCs are induced to consume their energy stores.

## DISCUSSION

The interest on the role of CSCs is increasing due to their involvement in tumor drug resistance and relapse. As in many cancers, CSCs were identified/isolated also in GB [37], the most malignant and aggressive of brain tumors [1]. It is known that GSCs are a heterogeneous population present in both intratumoral perivascular and necrotic/hypoxic niches. These microenvironments, maintain stemness, regulate proliferation, self-renewal and fate and protect them from environmental insults [2] resulting from the imbalance between the tumor growth and the vascular expansion that induces metabolic stress and O_2_ gradient throughout the tumor mass, with a very low O_2_ pressure within the intratumoral tissue [38]. Hypoxia plays a crucial role in stem cell biology acting through HIF-1α, responsible for many aspects of malignant progression, being involved in the adaptive response to the environmental changes [39, 7]. The role of PPARs in the regulation of glucose and lipid metabolism has been studied in different cellular models including brain cells [40-41, 21], the highest glucose and ATP consumer of all cells. Although the expression pattern of PPARs has been described in several tumors including gliomas [15], their role in tumorigenesis is still debated.

Therefore we evaluated the presence of PPARs, particularly α and γ isotypes, in GSCs, isolated from GB specimens exposed to hypoxia. We demonstrate that hypoxia upregulates HIF-1α protein and enhances GSC proliferation and induces high levels of PPARα, even increased with respect to normoxic cells. The nuclear localization of PPARα suggests that it is transcriptionally active and influences GSCs proliferation, as observed also in breast cancer cell lines [24]. On the contrary, PPARγ is downregulated, although not significantly, in hypoxia with a prominent cytoplasmic localization.

To correlate the expression of these receptors to the peculiar glucose and lipid metabolism of GSCs, their glycogen and lipid stores were analyzed, in normoxic and hypoxic conditions. Despite its relatively low levels in the adult brain, as compared to peripheral tissues, glycogen is the largest energy reserve of the CNS. It represents an advantageous form of glucose storage, as it can be rapidly mobilized without requirements for ATP and, differently from fatty acids, it can yield ATP under anaerobic conditions. In this way glycogen metabolism ensures protection against hypoglycemic neural injury and maintains neurons and axons function during intense periods of activation [42]. The observation of glycogen storages in GB neurospheres is a novel finding for these cells. The granules were found to be more abundant in hypoxia than in normoxia as also confirmed by the increased number of glycogen-positive cells in hypoxic condition. These results suggest that hypoxia may trigger glucose storage in glycogen. In fact, as previously demonstrated both in non malignant and in cancer cells, the hypoxia may act, in an early pro-survival condition, as a warning signal for cell to anticipate the extreme conditions [43]. Glycogen metabolism may represent a survival pathway, by which cells can accumulate energy reserve that can be quickly utilized under more drastic, nutrient-limiting conditions [44]. Parallel findings showed that hypoxia, through HIF-1α, induces the expression of genes encoding the enzymatic proteins required to convert glucose to glycogen, such as glycogen synthase [43-46]. The evaluation of the expression of the active and inactive form of GSK3β, involved in the regulation of glycogen synthase activity, demonstrates that the active form of the protein is downregulated, while its inactive form is upregulated in hypoxic condition. It is conceivable that hypoxia, by promoting the phosphorylation and inactivation of the GSK3β, may enhance the expression and/or the activity of glycogen synthase. GSCs express the high affinity glucose transporter, GLUT-3 that is increased in hypoxia. In fact, it is shown that HIF-1α regulates the expression of GLUT-3 [47] and that their coexpression in glioma is significantly correlated with the tumor pathological grade [48]. Therefore, hypoxia may directly regulate cellular glycogen storages by increasing the expression of glucose transport also in GSCs. Through this mechanism GSCs manage to have access to nutrients even in hostile microenvironment, driving tumor growth and survival [35, 49]. It is noteworthy in this context to recall that: i) PPARα is highly expressed in GB cells, paralleling malignancy grade [30]); ii) treatment of Aβ-loaded hippocampal neurons with a PPARα agonist, beside improving their survival, increases β-catenin stabilization, thus suggesting activation of Wnt canonic pathway [50]; iii) recent paper from Cisternas et al. [51] demonstrates that this pathway is involved in glucose utilization and glycolitic metabolism. Thus, it is tempting to speculate that low O2 and glucose concentrations, existing inside GB mass, induce an adaptive response involving also PPARα, leading to glucose and fatty acid utilization as energy fuels for GSC survival.

Since several studies reported that lipid levels in malignant gliomas are higher than normal tissues [14, 30, 52], the presence of lipid stores in the form of LDs was studied. Massive LD accumulation in GSCs was observed under hypoxic conditions, as also described in human hypoxic mesenchymal stem cells, exhibiting adipocyte-like phenotype with cytoplasmic accumulation of lipids [52]. Moreover, in hypoxic neurospheres, we observed upregulation of perilipin, possibly induced by PPARα, similarly to human hepatocytes treated with PPARα agonists [34].

This paper demonstrates that GSCs proliferation and survival and their metabolic signature are PPARα dependent. In fact, a specific antagonist of this isotype, GW6471, negatively affected cell proliferation and induced apoptosis, leading to reduced number and sizes of spheres. The hypoxic neurospheres were more resistant to the treatment than normoxic ones and required higher concentration of the antagonist. We interpreted this result on the basis that the higher levels of PPARα in hypoxic neurospheres together to hypoxia itself may contribute to this resistance. As a whole, our data confirm that GW6471 has a cytostatic and cytotoxic effect in GSCs, as in breast and renal cancer cell lines [24-25], and suggest that PPARα may control GSCs proliferation and survival. Notably, our results suggest that PPARα is involved in the process of synthesis and demolition of glycogen as its inhibition determines a decrease of its content. While in normoxia the reduction of glycogen content may be mainly due to an increase of glycogenolysis, in hypoxia it may be dependent on both the blockade of glycogen synthesis and the increase of its demolition. The demonstration that glycogen synthase-2 is a PPARα target gene in rat and mouse primary hepatocytes [53] and that the synthesis of glycogen is affected in PPARa^-/-^ mice [54] support our hypothesis. It is noteworthy that both Hexokinase II and PKM2, target genes of HIF-1α, are upregulated in hypoxia with respect to normoxia. These data suggest that GSCs have an active glycolysis in hypoxia, thus supporting both the bioenergetics and biosynthetic pathways [52]. In addition, hexokinase, other than to be involved in glycolysis and glycogen synthesis, has a role in protecting cells against apoptosis [55]. Our results show that the hypoxic neurospheres are less prone to apoptosis upon GW6471 treatment, being endowed with higher HEKII levels.

Interestingly we observed that GSCc, which express high levels of GLUT-3 in hypoxia, uptake more glucose in this condition than in normoxia and that the antagonist treatment reduces the influx of glucose in both conditions. Therefore we hypothesize that the reduced uptake of glucose, may be due to the reduced expression of GLUT-3, and may be correlated to the inhibition of PPARα. GW6471 indeed seems to force cells to consume their glycogen reservoir.

Several findings report that hypoxia induces the expression of genes involved in fatty acid uptake and intracellular lipid accumulation and impairs cholesterol metabolism [9, 56]. Particularly, FABP7, involved in fatty acids uptake, is induced by HIF-1α, leading to the accumulation of LDs and contributing to cancer cell growth [33]. Accordingly, our results show enhanced expression of FABP7, accumulation of LDs and increase of GSCs proliferation in hypoxia. In this condition, PPARγ, a regulator of adipogenesis, did not significantly change, thus resulting not involved in lipid accumulation as described in mesenchymal stem cells, where the hypoxic stimulus did not induce the expression of genes markers of mature adipocytes, including PPARγ [57]. Our results are also in agreement with Bensaad et al. [56] demonstrating that lipid storage in hypoxia is due to fatty acid uptake, rather than to *de novo* lipid synthesis.

As regard cholesterol content we found that hypoxic cells exhibited less cholesterol than normoxic ones, although the levels of mRNA of MVA enzymes and of LDL-receptor are not altered in both conditions. A possible explanation is that LDs in hypoxic GSCs may contain intermediates of mevalonate pathway such as squalene, lanosterol and lathosterol, as observed in a hypoxic GB cell line, where these intermediates accumulate since their conversion to cholesterol is oxygen-dependent [58]. In addition, also LDs of mutant yeast strains, used as a model for anaerobic growth, consist of squalene together with triacylglycerols and sterylesters. Both in yeasts and in mammalian cells the stored squalene is involved in clustering and distribution of LDs [58].

GW6471 treatment negatively affected the content of LDs, FABP-7, MVA enzymes, LDLR, cholesterol and cell proliferation. In normoxia, the antagonist reduced the levels of LDs by inhibiting the synthesis and uptake of cholesterol rather than fatty acid influx, since FABP7 levels did not change. Conversely, in hypoxia, the treatment affected both cholesterol and triglyceride content, blocking the MVA pathway and decreasing the levels of LDL receptor and FABP7. Therefore, we propose that the composition of LDs may be different in normoxic and hypoxic neurospheres. As regard mRNA for LDLR, the treatment was effective both in hypoxia and normoxia, downregulating the receptor and impairing the intracellular influx of cholesterol needed for cell membrane turnover and for LD storage.

Since MVA pathway is crucial for GB cell survival and growth, its inhibition by the PPARα antagonist contributes to cell death induction, cell cycle arrest and repression of cancer cell migration [59]; in fact, statins commonly used in the treatment of hypercholesterolemia, were also used to inhibit cancer [60]. Our results indicate that the inhibition of PPARα by GW6471 may produce effects similar to statins, down-regulating MVA pathway and up-regulating PPARγ. The association of statins and PPARγ agonists were largely used for cancer treatment [60]

All the above results were obtained with GSCs grown in high glucose levels. Noteworthy, patients with GB are at particular risk of hyperglycemia, because their peritumoral edema is regularly treated with high-dose of glucocorticoids, which are known to increase plasma glucose [61]. Hyperglycemia, by activating various signaling pathways that control cancer cell behavior such as proliferation, migration, invasion and recurrence [62] and providing an extra energetic fuel, promotes tumor growth [63] by supporting the high glycolytic metabolism of GB. In this paper, we also compared hypoxic GSCs grown in standard culture conditions to hypoxic GSC grown in restricted glucose concentration, which is more representative of the interstitial fluid in solid tumors, thus mimicking the *in vivo* situation. Indeed, tumor microenvironment associated with hypoxia and nutrient restriction may be important in the regulation of tumor progression.

It is known that, as result of the inhibition of angiogenesis, cancer cells exposed to both hypoxia and starvation, are induced to be more aggressive [64]. Differently to hypoxia, nutrient depletion has not been well investigated in terms of its effect on cancer stem cells. Nutrient limitations within solid tumors may require that malignant cells exhibit metabolic flexibility to sustain growth and survival [65].

On the basis of these considerations, we investigated the impact of glucose shortage on proliferation and metabolic pattern of hypoxic neurospheres and the effect of GW6471 on GSCs growth/survival in restricted glucose levels. We confirm that glucose restriction contributes to tumor progression since the GSCs grown at low glucose levels were higly proliferating and paralleled by a significant increase of PPARα and decrease of PPARγ, thus confirming the opposite role of the two nuclear transcription factors in GSCs.

It is intriguing to underline that glucose metabolic rate and uptake did not correlate with GLUT3 expression but with cell proliferation. In fact, GLUT3 did not significantly change, switching from high (3,15g/L) to restricted (0.25g/L) glucose. In response to glucose reduction, GSCs utilize their glycogen storage to support metabolic and survival pathways in stressful conditions [43-44]. Moreover, GSCs grown in restricted nutrients decreased their LD content and increased their cholesterol content to sustain the high cell proliferation rate. We suggest that, since the uptake of fatty acids was not influenced by the different glucose concentrations, the upregulation of PPARα in the restricted glucose condition may also lead to the induction of the β-oxidation of fatty acids producing acetyl-CoA to be utilized for the synthesis of cholesterol. In this condition GW6471 induces, similarly to what observed in high glucose condition, an inhibitory effect on cell proliferation and on glycogen, LDs and cholesterol content.

In conclusion, our data support, for the first time, a crucial role of PPARα in regulating the metabolic switch that allows GSCs to survive in O2 and nutrient limitations and demonstrate the effectiveness of GW6471 as anti-tumor and lipid/glucose storage-reducing drug. Targeting specific metabolic pathways essential to GSCs survival is a new and promising approach against cancer. We here propose the use of GW6471 as a potential novel strategy for targeting GSCs to be associated with the gold standard protocols.

## MATERIALS AND METHODS

### Sample classification

GSCs were isolated from post-surgical specimens of patients with diagnosis of Grade IV glioma, from S. Salvatore Hospital, L’Aquila, Italy. All samples were classified according to the World Health Organization guidelines (WHO). This study was ethically approved (Hospital Ethics Committee), and all patients were voluntary signing an informed consent

### Neurospheres primary culture

Tumor samples, were dissected, dissociated, incubated in HBSS solution containing 0.125% Trypsin, 0.125% EDTA and 0.2mg/ml DNAse (Sigma Chemical St. Louis, CO), at 37°C, for 20 min and cultured, until formation of primary tumorsphere, in Dulbecco’s modified Eagle’s medium/F-12 medium (DMEM-F12, Sigma), containing 100 units /ml penicillin/streptomycin, 2mM glutamine (Sigma), 2% B27 supplement (Invitrogen Corporation, CA, USA) and 20ng/mL of both epidermal growth factor (EGF) and fibroblast growth factor (FGF2) (Peprotech, Rocky Hill, NY), at 37°C in humidified 95% air-5% CO_2_ atmosphere.

Primary spheres were dissociated mechanically and plated at a density of 2500 -5000 cells/cm^2^ for several passages (clonal selection).

Isolated neurosheres were assayed for their stemness properties in terms of immunopositivity to stem cell markers by immunofluorescence and cytofluorimetry.

### Hypoxia

Hypoxia was performed into a hypoxic chamber, Galaxy 14S CO2 incubator, (New Brunswick Scientific, Edison, USA) with a gas mixture of 94% N_2_, 5% CO_2_ and 1% O_2_, for 72hr, at 37°C.

### Cell proliferation by BrdU assay

Neurospheres proliferation was assayed by the incorporation of BrdU. Single cells from neurospheres (5x10^3^ cells/well) were grown for 5-7 days and incubated with BrdU labeling and detection kit III (Roche, Basel, Switzerland) for 72hr. following the manufacturer’s instructions. The absorbance of the samples (405 nm) was measured with a spectrophotometric microplate reader (Infinite F200 Tecan, Switzerland).

### Immunofluorescence

Neurospheres were allowed to adhere on poly-L-lysine coated glass coverslips (15µg/ml), fixed with 4% paraformaldehyde in PBS, for 10 min, at RT and incubated with following antibodies: monoclonal anti-nestin (1:100, Chemicon International, Temecula, CA, USA), anti-CD133/1 (1:50, Miltenyl Biotec. Inc., Auburn, CA, USA), anti-β-tubulin III (1:500, Promega, Mannheim, Germany), polyclonal anti-SOX2 (1:500, Abcam) and anti-GFAP (1:200, Sigma), rabbit anti-HIF-1α (1:200, Santa Cruz, CA, USA), anti-PPARɑ and anti-PPARγ (1:400, Thermo Scientific Inc., USA), anti-GLUT3 (1:100, Abcam, Cambridge, UK) and mouse IgM anti-glycogen (1:400, generous gift from Prof. Yoshinobu Baba, Nagoya University). Primary antibodies were revealed by Alexa Fluor 488 or 633 anti-rabbit IgG or Fluorescein Isothiocyanate (FITC) anti-mouse IgM secondary antibody (Sigma).

For bodipy staining, neurospheres were incubated with 4,4-difluoro-1,3,5,7,8-pentamethyl 4-bora-3a,4a-diaza-sindacene (1µg/mL, BODIPY 493/503 Molecular Probes, Invitrogen), for 10 min, at RT. Coverslips were mounted with Vectashield Mounting Medium with Dapi (Vector Laboratories, Burlingame, CA, USA) and examined at a Leica TCS SP5 confocal microscope (Mannheim, Germany).

### Cytofluorimetric analysis

#### Flow cytometry analysis of neurospheres cultures

The dissociated neurospheres were examined by FACSCalibur flow cytometry (BD Instruments Inc., USA) for detection of following markers: β-tubulin III (Alexa Fluor 488), anti-SOX-2 (PE Mouse), anti-nestin (Alexa Fluor 647) — all acquired from Becton Dickinson (BD) — anti-GFAP (Glial fibrillary acidic protein, Sigma-Aldrich) and anti-CD133 (anti-PROM-1, Abnova).

The single-cell suspensions (0.5x10^6^ cell/tube) were washed, fixed, for 15 min at RT, with 2% formaldehyde in PBS and permeabilized for 5 min with Triton X-100 at RT for intracellular markers. Cells were washed with PBS and then incubated, for 1h, at RT, with selected primary antibodies for 1h at RT in the dark. For CD133 and GFAP detection, secondary PE (Abcam) and FITC-conjugated (Millipore) antibodies respectively were used for 1h at RT. The population of interest was gating according to its Forward Scatter (FSC)/Side Scatter (SSC) criteria. 10,000 events were acquired for each sample and analyzed by CellQuest software (BD Instruments Inc., USA).

### Glycogen content

Single cells from neurospheres were fixed with 2% formaldehyde in PBS, for 15 min, at RT and permeabilzed with TX-100 0.1% (Sigma). Cells were incubated with mouse IgM anti-glycogen (1:200) antibodies for 1h, at RT, recognized by FITC anti-mouse IgM secondary antibodies and evaluated using a FACS Calibur cytometer analyzer (Becton Dickinson, San Diego, CA, USA). Data were analyzed using CellQuest software (Becton Dickinson).

### PPARα antagonist treatment

BrdU labeled cells were treated with different concentrations of GW6471 (2-16µM, fc), for 72hr and incubated in normoxia and hypoxia respectively. For immunofluorescence, western blotting and RT-PCR analyses cells were treated with 8µM in normoxia and 8-16µM GW6471 in hypoxia for 72hr.

### Apoptosis

Normoxic and hypoxic neurospheres, treated with 8-16µM GW6471 for 72hr, were processed by Cell Death Detection ELISA^PLUS^ kit (Roche) following the manufacturer’s instructions.

### Neurospheres number and size

For the analysis of spheres formation, single cells from neurospheres were treated with with 8μM GW6471 in normoxia and 16μM dose in hypoxia, for 72h. After treatment, formed spheres were photographed and counted at contrast phase microscopy.

### Western blotting

Normoxic and hypoxic neurospheres treated or not with GW6471 were lysated in RIPA buffer, sheared through a 22-gauge needle and centrifuged at full-speed (Eppendorff microfuge 5418), at 4 °C, for 30 min. Proteins were assayed by the bicinchoninic acid protein assay kit (Pierce, Rockford, IL, USA). For glucose transporter, GLUT3, lysates of cell membranes were used as antigen.

Protein lysates (30-50 µg/lane) were separated on 10% SDS polyacrylamide gel and electroblotted onto polyvinyldifluoride membrane (Sigma). Non specific binding sites were blocked by 5% non fat dry milk (Bio-Rad Laboratories, Hercules, CA, USA) in Tris-buffered saline (TBS; 20 mM Tris-HCl, pH 7.4, containing 150 mM NaCl), overnight at 4 °C. Blots were probed with following primary antibodies: rabbit polyclonal anti-HIF-1α (1:200), anti-perilipin A (1:500), anti-*adipophilin* (1:500), anti-actin (1:2000) (Sigma), anti-glycogen synthase kinase-3β phospho-ser9 (pGSK3β, 1:500, Enogene Biotech., Aachen, Germany), anti-glycogen synthase kinase-3β (GSK3β, 1: 5000, Abcam), anti-GLUT3 (1:5000, Abcam), anti-pyruvate kinase isoenzyme type M2 (PMK2, 1:1000, Abcam), anti-hexokinase II (1:500, Cell Signaling Technology Inc., Danvers MA, USA), anti-fatty acid binding protein 7 (FABP7, 1:500 GeneTex Inc., Irvine, CA, USA) and mouse monoclonal anti-PPARα (1:200) and anti-PPARγ (1:500) (Novus Biologicals, Littleton, CO, USA), anti-glycogen phosphorylase isoenzyme BB (GPBB,1:1000, Abcam) overnight, at 4°C. All antibodies were diluted with TBS containing 0.25% Tween-20 (TTBS) and 5% non fat dry milk. As secondary antibodies, peroxidase conjugated anti-rabbit or anti-mouse IgG (1:10000, KPL, Gaithersburg, USA), in TTBS containing 5% non fat dry milk, were used for 1 h, at RT. Immunoreactive bands were visualized by enhanced chemiluminescence (Cyanagen S.r.l, Bologna, Italy), according to the manufacturer’s instructions.

### Glucose uptake

Glucose uptake was evaluated using a fluorescent D-glucose analog 2- [N-(7-nitrobenz-2-oxa-1,3-diazol-4-yl)amino]-2-deoxy-D-glucose (2-NBDG Molecular Probe, Invitrogen) as described by [66-67], with some modifications. Neurospheres treated or not with 8µM GW6471 in normoxia and with 8-16µM GW6471 in hypoxia, were incubated with complete DMEM-F12 without glucose (Biowest, Nuaille’, France), for 1h, at RT (starvation) and incubated with 200µM 2-NBDG, for 1h, at 37°C. Control were performed incubating neurospheres with 200µM 2-NBDG in presence of DMEM-F12 containing glucose. After washing with PBS spheres were fixed with 4% paraformaldehyde in PBS, for 10 min, at RT. Coverslips were mounted with Vectashield Mounting Medium with Dapi (Vector) and examined at a Leica TCS SP5 confocal microscope.

### Quantitative real time-PCR (qRT-PCR)

Neurospheres treated or not with 8µM GW6471 in normoxia and with 8-16µM in hypoxia were harvested with Trizol (Invitrogen) and total RNA was isolated using the Nucleo Spin RNA II kit (Macherey-Nagel) according manufacturer’s instructions. cDNA was transcribed using Super Script II Reverse Transcriptase (Invitrogen), starting from 0.5 micrograms of high pure RNA. Mevalonate genes expression profiles were evaluated with specific primer sets (Table 1) and using Sso Fast Eva Green reagents (Bio-Rad), β2-microglobulin was used as housekeeping gene. qRT-PCR protocol was: a pre-heating step for 3 min, at 95°C, 40 cycles at 95°C for 10 seconds and 60°C for 30 seconds and last end-step at 65°C for 10 seconds. Results were analyzed with 2-^ΔΔCt^ method [68].

**Table 1:** Specific primer sets

RT-PCR primers		
Gene symbol	Forward Primer	Reverse Primer
HMGCR	5’-taccatgtcaggggtacgtc-3’	5’-ccagtcctaatgaaaccttagaag-3’
MVK	5’-gctcaagttcccagagatcg-3’	5’-atggtgctggttcatgtcaa-3’
FDPS	5’-agcaggatttcgttcagcac-3’	5’-tcccggaatgctactaccac-3’
FDFT1	5’-ggtcccgctgttacacaact-3’	5’-aaaactctgccatcccaatg-3’
RABGGTA	5’-gaccccctgctgtatgagaa-3’	5’-cacctcggcatactccatct-3’
LDLR	5’-gaatttggccagacacaggt-3’	5’-caccgtacccagctgatttt-3’
β2M	5’-cctggattgctatgtgtctgg-3’	5’-ggagcaacctgctcagataca-3’

### Cholesterol assay

Neurospheres treated or not with 8µM GW6471 were extracted with chloroform:isopropanol:NP40 (7:11:0.1) and the organic phase was dried at 50C° and put under vacuum for 30 min. Dried lipids were dissolved with Cholesterol Assay Buffer and assayed according to manufacturer’s instructions (Cayman, Michigan, USA). The fluorescence of the samples was measured at Ex/Em = 535/590 nm with a microplate reader (In?nite F200 Tecan, Switzerland).

### Cell proliferation by carboxyfluorescein diacetate succinimidyl ester (CFSE)

Cell proliferation is evaluated by the CellTrace™ CFSE cell proliferation kit (Molecular Probes, Life Technology, Carlsbad, CA, USA), according to manufacturer’s protocol.

Neurospheres were allowed to adhere on poly-L-lysine coated glass coverslips and fixed in 4% paraformaldehyde in PBS, for 10 minutes, at RT. Coverslips were mounted with Vectashield Mounting Medium and examined at a Leica TCS SP5 confocal microscope.

### Glycogen assay

Neurospheres grown in 0.25g/L glucose,and 16µM GW6471 treated were homogenized with ice cold glycogen hydrolysis buffer, for 10 min, centrifuged at 12000 rpm, for 5 min and supernatants were assayed according to manufacturer’s instructions (BioVision, Milpitas, CA, USA). The product was revealed at 450nm.

### Statistical analysis

For statistical analysis samples were processed by SPSS software and analyzed by ANOVA test followed by Scheffe’s post hoc test” analysis. *P < 0.05; **P < 0.005, ***P < 0.0005. All data are mean ± SD of three separate experiments run in triplicate..

## SUPPLEMENTARY MATERIALS FIGURES


