# Supplementary material for: Energy metabolism in glioblastoma stem cells: PPARα a metabolic adaptor to intratumoral microenvironment
Source: Oncotarget. 2017 Jul 7;8(65):108430–50. doi: 10.18632/oncotarget.19086 (PMC5752454; doi:10.18632/oncotarget.19086)
Supplement: Supplementary file 1 [file oncotarget-08-108430-s001.pdf]

Energy metabolism in glioblastoma stem cells: PPARα a metabolic adaptor to intratumoral microenvironment

SUPPLEMENTARY MATERIALS

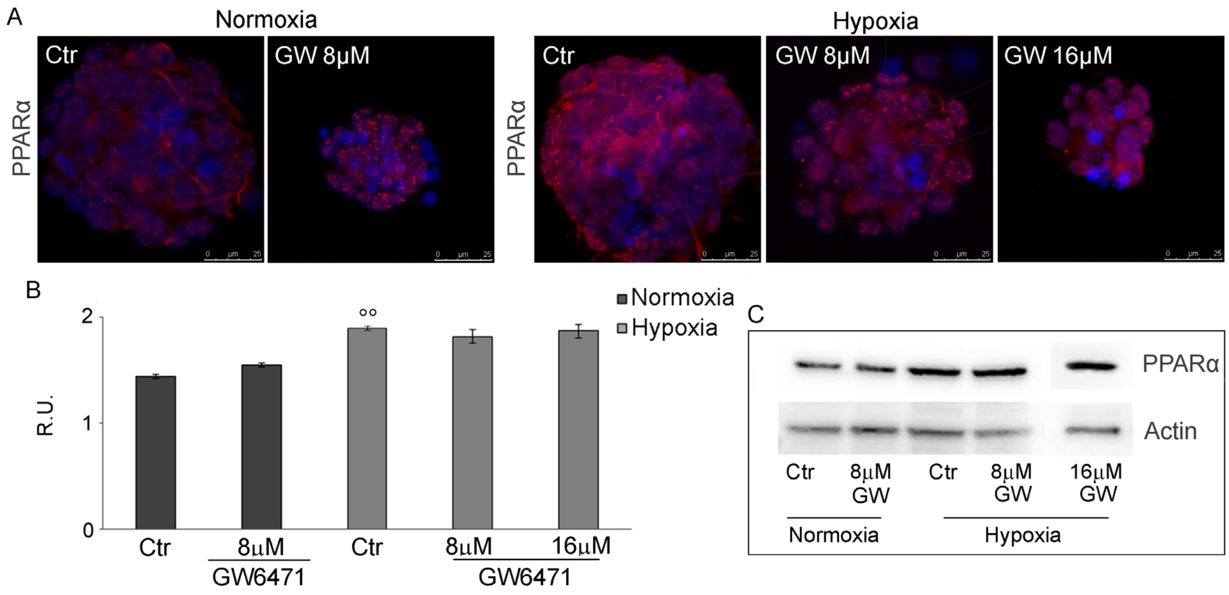

Supplementary Figure 1: (A) Immunofluorescence of PPARα in GSCs treated with 8μM GW6471 in normoxia and 8-16μM in hypoxia, for 72h shows an increase of PPARα in hypoxic cells, while the treatment did not affect PPARα expression**.** Nuclei are stained with Dapi. Bar =25μm. (**B**) Densitometric and western blotting (**C**) analyses for PPARα upon GW6171 treatment in normoxic and hypoxic neurospheres, confirmed the immunofluorescence data. Data are means ± SD of three different experiments. °° *P*<0.001.

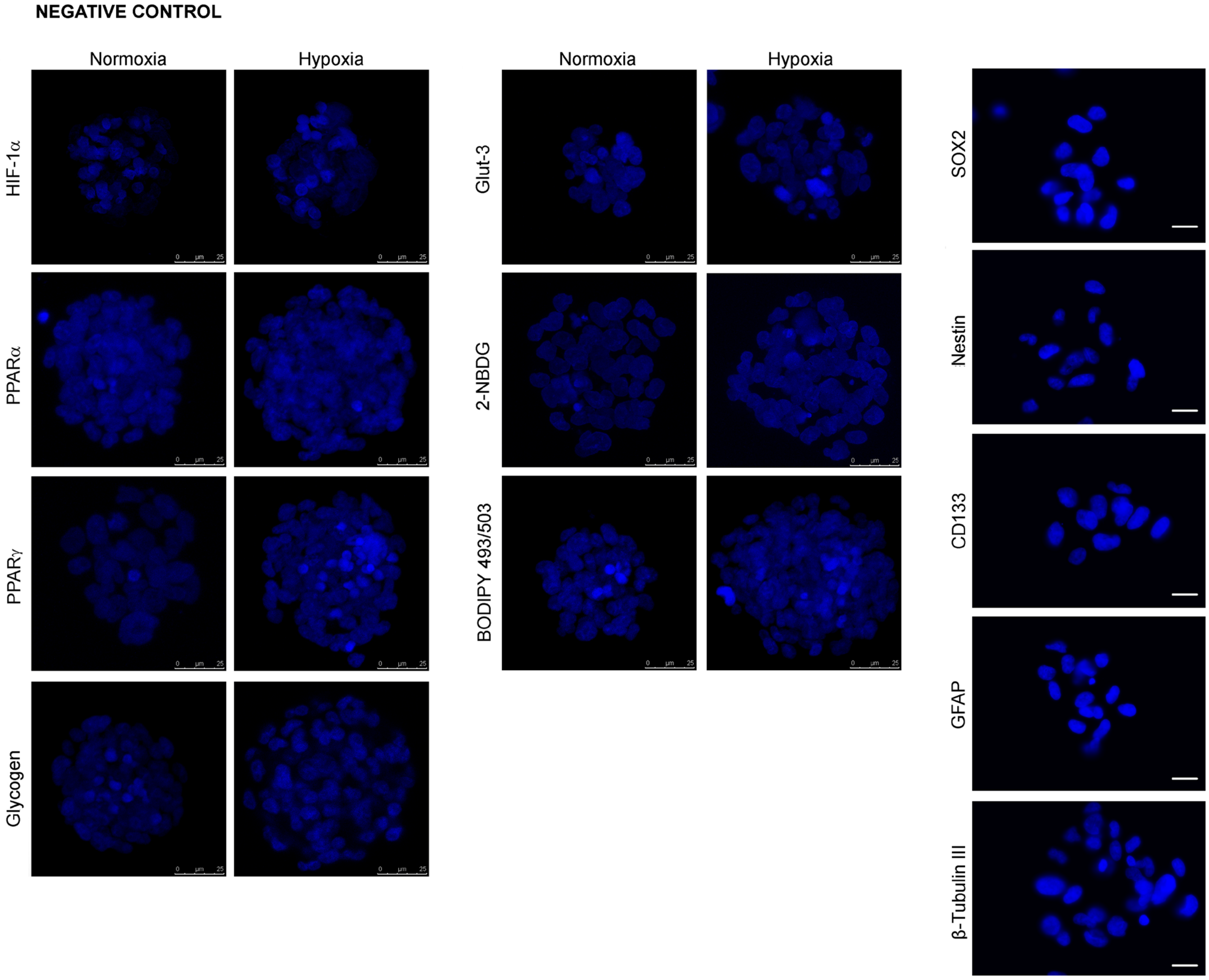

Supplementary Figure 2: Negative controls for all the immunofluorescence data presented in the ms. Nuclei are stained with Dapi.
